# Unilateral Cervical Nodal Metastasis Is an Independent Prognostic Factor for Esophageal Squamous Cell Carcinoma Patients Undergoing Chemoradiotherapy: A Retrospective Study

**DOI:** 10.1371/journal.pone.0101332

**Published:** 2014-06-30

**Authors:** Peng Zhang, Mian Xi, Lei Zhao, Qiao-Qiao Li, Liru He, Shiliang Liu, Jingxian Shen, Meng-Zhong Liu

**Affiliations:** 1 Sun Yat-sen University Cancer Center; State Key Laboratory of Oncology in South China, Collaborative Innovation Center for Cancer Medicine; Department of Radiation Oncology, Cancer Center, Sun Yat-sen University, Guangzhou, People’s Republic of China; 2 Sun Yat-sen University Cancer Center; State Key Laboratory of Oncology in South China, Collaborative Innovation Center for Cancer Medicine; Imaging Diagnosis and Interventional Center, Cancer Center, Sun Yat-sen University, Guangzhou, People’s Republic of China; Ospedale Pediatrico Bambino Gesu’, Italy

## Abstract

**Purpose:**

To determine the prognostic significance of unilateral cervical lymph nodal metastasis (CLNM) in patients with inoperable thoracic esophageal squamous cell carcinoma (SCC) and to identify significant prognostic factors in these patients.

**Patients and methods:**

This retrospective study involved 395 patients with inoperable esophageal SCC treated with concurrent chemoradiotherapy. The patients were classified into three groups according to their cervical lymph node status: group A, no evidence of CLNM; group B, unilateral CLNM; group C, other distant metastases. Overall survival (OS) and progression-free survival (PFS) were calculated. Significant prognostic factors were identified using univariate and multivariate analyses.

**Results:**

The 3-year OS rates in groups A, B and C were 46.7%, 33.5% and 8.3%, respectively (*p*<0.001, log-rank test). The corresponding PFS rates were 40.7%, 26.4% and 4.7% (*p*<0.001, log-rank test). Group B had a similar prognosis to that of group A and better 3-year OS (*p* = 0.009) and PFS (*p* = 0.006) rates than those of group C. Multivariate analysis demonstrated that T stage, chemotherapy regimen and cervical lymph node involvement were independent prognostic factors affecting OS and PFS.

**Conclusions:**

Compared to other distant metastases, unilateral CLNM is associated with longer OS in esophageal SCC and should be regarded as a regional disease. Sex, T stage, concurrent chemotherapy modality and cervical lymph node involvement are independent predictors of survival in esophageal SCC.

## Introduction

Esophageal cancer is one of the most life-threatening tumors, with a 5-year survival rate of only 17% [1]. The prognosis of patients with distant metastases is even more disappointing. However, numerous studies have shown that some patients with thoracic esophageal squamous cell carcinoma (SCC) and cervical lymph node metastasis (CLNM) could have better long-time survival than patients with visceral metastasis, which suggests that CLNM should be regarded as regional spread rather than distant metastasis [2], [3], [4]. The Japanese Society for Esophageal Diseases (JSED) has divided cervical nodes into four groups: cervical paraesophageal nodes, deep cervical nodes, retropharyngeal nodes and supraclavicular nodes, and involvement of the cervical paraesophageal nodes was defined as stage N1 in the case of cancers of the upper third of the esophagus [5]. Moreover, the American Joint Committee on Cancer (AJCC 7^th^ edition) defined cervical paraesophageal nodes as regional nodes in the case of esophageal cancer [6]. According to the Chinese non-operative stage of esophageal cancer, patients with CLNM are considered to be in stage N1 (cervical esophageal cancer) or N2 (thoracic esophageal cancer) [7]. However, the prognostic significance of unilateral CLNM in esophageal cancer, if any, has not yet been explored in detail. The purpose of this study is to determine whether unilateral CLNM has an impact on the prognosis of patients with inoperable esophageal SCC and to analyze prognostic factors for esophageal SCC.

## Patients and Methods

### Ethics statement

This study was approved by the institutional review board (IRBs) of Cancer Center, Sun Yat-sen University. Written informed consent was obtained from all the patients in accordance with the regulations of the IRBs.

### Clinical data and patient groups

We retrospectively studied 395 consecutive patients who were diagnosed with esophageal SCC and treated with concurrent chemoradiotherapy in Sun Yat-sen University Cancer Center between February 2002 and December 2011. These patients had either refused surgery or were unable to undergo surgery. The pretreatment work-up included complete history collection, physical examinations, computed tomography (CT) scans of the chest and abdomen, barium esophagography and endoscopic ultrasonography. In this study, we employed the AJCC staging system (6^th^ edition). In all patients, the diagnosis was pathologically confirmed to be SCC. The date of last follow-up was April 1^st^, 2013, and the median follow-up time was 35 months (range, 10–117 months).

The 395 study patients were classified into three groups depending on the extent of regional/metastatic spread at the time of initial diagnosis: group A, no evidence of CLNM (n = 204); group B, unilateral CLNM (n = 106); and group C, other distant metastases (n = 85).

### Radiotherapy and chemotherapy

External beam radiotherapy was administered using 6–10 MV X-rays. All patients received three-dimensional conformal radiotherapy at a dose of 1.8–2.0 Gy per fraction, five times a week. The patients underwent radiotherapy for 4–6 weeks, receiving a total dose of 46–70 Gy. The primary gross tumor volume (GTV) and the volume of involved lymph nodes (GTV-N) were determined. The conformal clinical target volume (CTV) included the GTV with a 3-cm margin in the craniocaudal direction and a 0.5-cm margin in the lateral and anteroposterior directions. The CTV of SCCs involving the upper third of the esophagus encompassed the right and left supraclavicular regions. In patients with unilateral CLNM, the contralateral supraclavicular fossa was included in the CTV for prophylactic purposes. The CTV for lymph nodes included the GTV-N without an additional margin. The planning target volume included the CTV with a 1-cm margin in the superior–inferior direction and a 0.5-cm margin in the lateral direction [8].

Concurrent chemotherapy was administered using regimens that mainly included cisplatin plus 5-fluorouracil and cisplatin plus docetaxel. In all, 116 patients were treated with two cycles of 60 mg/m^2^ docetaxel and 80 mg/m^2^ cisplatin delivered on days 1 and 22 of radiotherapy [9]. In all, 51 patients received at least four cycles of docetaxel (30 mg/m^2^) and cisplatin (35 mg/m^2^) per week. Another 129 patients were treated with two cycles of 60 mg/m^2^ cisplatin administered on days 1 and 29 and 300 mg/m^2^/24 h 5-fluorouracil administered on days 1–3 and days 29–31 [8]. The remaining 99 patients received other chemotherapy regimens such as navelbine plus cisplatin.

### Statistical analysis

Overall survival (OS) and progression-free survival (PFS) were the study endpoints. OS was calculated from the day of diagnosis to the date of death or censored at the date of last follow-up. PFS was computed from the day of diagnosis to the detection of recurrent disease or to the date of death in patients without evidence of disease recurrence, censoring at the date of last follow-up. The survival analysis was performed by the Kaplan–Meier method, and differences between the curves were analyzed using the log-rank test. As categorical data, sex (male vs. female), pathological grade, tumor location (upper third vs. middle third vs. lower third), primary tumor length (<3 cm vs. ≥3 cm, ≤6 cm vs. >6 cm), clinical T stage (T1/T2 vs. T3 vs. T4), clinical M stage (M0 vs. M1), cervical lymph node involvement, radiation dose (≤60 Gy vs. >60 Gy) and concurrent chemotherapy (DDP+5-Fu vs. DDP+docetaxel vs. others) were included in the log-rank test. The three categories of CLNM (group A, group B, group C) were analyzed using pairwise comparison, and the p value was set at 0.017 (0.05/3) according to Hochberg’s step-up procedure [10]. Multivariate analyses using the Cox proportional hazards model were used to calculate the hazard ratio and to test independent significance by backward elimination of non-significant explanatory variables. The criterion for statistical significance was set at α = 0.05, and *p* values were determined from two-sided tests. All statistical analyses were performed using SPSS 16.0 software (SPSS Inc., Chicago, IL).

## Results

### Patients and clinicopathological features

The demographic and clinical data of the patients are shown in Table 1. Of the 395 patients, 316 were men (80.0%), and 79 were women (20.0%). The median age of the patients was 58 years (range, 35–75 years). The primary tumors were located in the upper third of the esophagus in 154 patients (39.0%), in the middle third in 206 patients (52.2%) and in the lower third in 35 patients (8.8%). According to the AJCC staging system (6^th^ edition), 43 patients were diagnosed with stage I/II disease, and 161 patients were diagnosed with stage III disease. The remaining 191 patients were found to have stage IV disease. Of these 191 patients, 106 had unilateral CLNM, 33 had bilateral CLNM and 52 had other distant metastases.

**Table 1:** Demographic and pathological characteristics of the study population.

Characteristic	No. of patients	
Age (years)		
Median	58	
Range	35–75	
Sex		
Male	316	(80.0%)
Female	79	(20.0%)
Pathological grade		
Well differentiated	36	(9.1%)
Moderately differentiated	178	(45.1%)
Poorly/undifferentiated	97	(24.6%)
Unknown	84	(21.2%)
Location		
Upper third	154	(39.0%)
Middle third	206	(52.2%)
Lower third	35	(8.8%)
Primary tumor length		
Median (cm)	6.0	
<3 cm	21	(5.3%)
≥3 cm, ≤6 cm	220	(55.7%)
>6 cm	154	(39.0%)
T stage		
T1–T2	54	(13.7%)
T3	184	(46.6%)
T4	157	(39.7%)
M stage		
M0	204	(51.6%)
M1a	62	(15.7%)
M1b	129	(32.7%)
Stage		
I–II	43	(10.9%)
III	161	(40.8%)
IV	191	(48.4%)
Cervical nodal involvement		
CLNM (–)	204	(51.6%)
Unilateral CLNM	106	(26.8%)
Other M	85	(21.5%)
Radiation dose (Gy)		
≤60	314	(79.5%)
>60	81	(20.5%)
Concurrent chemotherapy		
PDD+5-Fu	129	(32.7%)
PDD+Docetaxel	167	(42.3%)
Others	99	(25.0%)

### Influence of cervical lymph node involvement on survival

**Figure 1:**
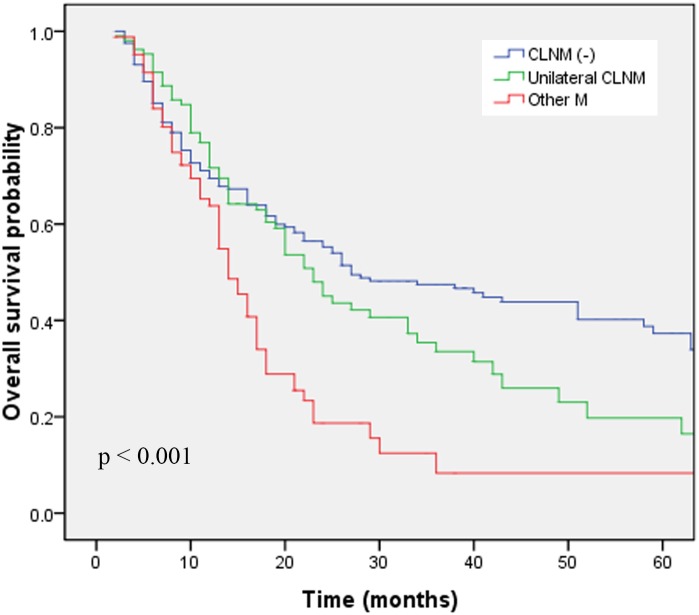
Overall survival in patients with different extents of cervical lymph node involvement.

**Figure 2:**
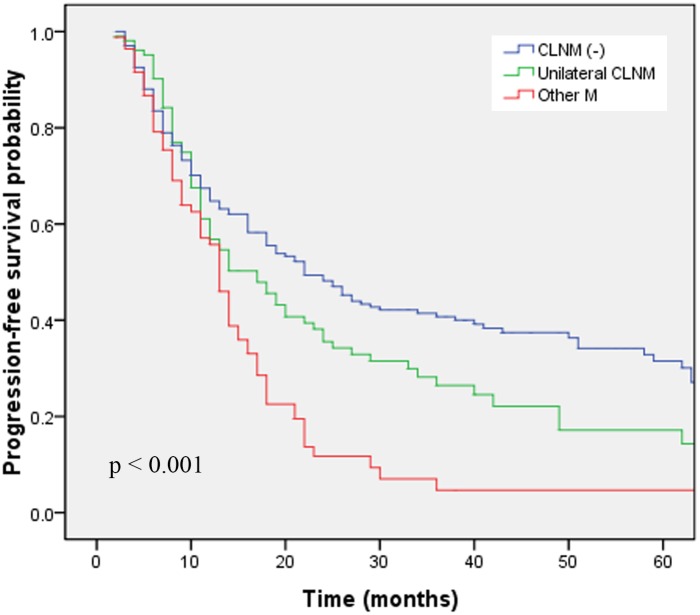
Progression-free survival in patients with different extents of cervical lymph node involvement.

By April, 2013, 235 of the 395 study patients had died. The 3-year OS rates in groups A, B and C were 46.7%, 33.5% and 8.3%, respectively (*p*<0.001; Fig. 1). The corresponding PFS rates were 40.7%, 26.4% and 4.7% (*p*<0.001; Fig. 2). The OS and PFS have been shown in Table 2. The 3-year OS rate was significantly higher in patients with CLNM than in patients with distant organ metastasis (*p* = 0.027). The laterality of CLNM did not have a significant impact on OS (*p* = 0.626) or PFS (*p* = 0.945). We next performed pairwise comparisons among the three groups. Group A and group B had a similar 3-year OS (*p* = 0.162) and PFS (*p* = 0.064). Group B had significantly better 3-year OS (*p* = 0.009) and PFS (*p* = 0.006) rates than those of group C. Group A had significantly better 3-year OS (*p*<0.001) and PFS (*p*<0.001) rates than those of group C.

**Table 2:** OS and PFS of esophageal SCC patients according to cervical lymph node involvement.

Group	No.	3 y OS	Median	95% CI	3 y PFS	Median	95% CI
			OS (m)			PFS (m)	
Group A	204	46.7%	34.0	13.1–54.9	40.7%	28.0	7.3–48.4
Group B	106	33.5%	27.0	13.2–40.8	26.4%	20.0	13.8–26.2
Group C	85	8.3%	23.0	18.9–27.1	4.7%	17.0	11.8–22.2

Patients with unilateral CLNM were further divided into three groups depending on the location of the primary SCC: (1) upper third of the thoracic esophagus (n = 41); (2) middle third of the thoracic esophagus (n = 58); and (3) lower third of the thoracic esophagus (n = 7). The 3-year OS rates were 39.7%, 28.9% and 19.0% while the 3-year PFS rates were 32.5%, 21.5% and 19.0% in patients with SSC of the upper, middle and lower thirds of the thoracic esophagus, respectively.

### Prognostic analysis

Table 3 shows the results of univariate analyses for each prognostic factor, namely, sex, pathological grade, primary esophageal tumor location, tumor length, clinical T stage, cervical lymph node involvement, clinical M stage, radiation dose and concurrent chemotherapy regimen. Univariate analysis showed that sex (*p*<0.001), primary tumor location (*p* = 0.009), tumor length (*p* = 0.008), clinical T stage (*p* = 0.001), clinical M stage (*p* = 0.001), cervical lymph node involvement (*p*<0.001) and concurrent chemotherapy regimen (*p* = 0.009) had a significant impact on OS. SCC of the upper third of the esophagus, female sex and the cisplatin plus docetaxel regimen were associated with significantly better OS than SCC of the middle or lower third of the esophagus, male sex and other chemotherapy regimens, respectively (Table 3).

**Table 3:** Univariate analyses demonstrating factors associated with OS and PFS.

Factor	No.	OS	PFS
		*p* value	*p* value
Sex		<0.001	0.006
Male	316		
Female	79		
Pathological grade		0.372	0.938
Well differentiated	36		
Moderately differentiated	178		
Poorly/undifferentiated	97		
Unknown	84		
Location		0.009	0.067
Upper third	154		
Middle third	206		
Lower third	35		
Primary tumor length		0.008	0.002
<3 cm	21		
≥3 cm, ≤6 cm	220		
>6 cm	154		
Clinical T classification		0.001	0.004
T1–T2	54		
T3	184		
T4	157		
Clinical M stage		0.001	<0.001
M0	204		
M1	191		
Cervical nodal involvement		<0.001	<0.001
CLNM (–)	204		
Unilateral CLNM	106		
Other M	85		
Radiation dose (Gy)		0.945	0.465
≤60 Gy	314		
>60 Gy	81		
Concurrent chemotherapy		<0.001	0.001
DDP+5-Fu	129		
DDP+Docetaxel	167		
Others	99		

To identify independent prognostic factors, the factors that were found to be significant on univariate analysis were subjected to multivariate analysis. Because there is a duplication between the M stage and cervical lymph node involvement, only cervical lymph node involvement was entered into the multivariate analysis. Multivariate analysis revealed that clinical T stage (*p* = 0.005), cervical lymph node involvement (*p*<0.001) and chemotherapy regimen (*p*<0.001) were independent factors affecting OS in esophageal SCC patients. Clinical T stage (*p* = 0.010), cervical lymph node involvement (*p*<0.001) and chemotherapy regimen (*p* = 0.002) were independent factors affecting PFS in esophageal SCC patients (Table 4).

**Table 4:** Multivariate analysis of factors influencing OS and PFS in esophageal SCC.

Endpoints	Variable	*P* value	HR	95% CI for HR
OS	Sex	0.002	0.615	0.450–0.840
	Location	0.071	1.214	0.983–1.499
	Tumor length	0.268	1.146	0.900–1.460
	T stage	0.005	1.248	1.070–1.455
	Cervical nodal involvement	<0.001	1.520	1.282–1.801
	Chemotherapy	<0.001	0.737	0.621–0.873
PFS	Sex	0.102	0.792	0.599–1.048
	Tumor length	0.145	1.183	0.944–1.482
	T stage	0.010	1.203	1.046–1.384
	Cervical nodal involvement	<0.001	1.495	1.277–1.750
	Chemotherapy	0.002	0.780	0.665–0.915

Abbreviations: 95% CI, 95% confidence interval; HR, hazards ratio; OS, overall survival; PFS, progression-free survival; SCC, squamous cell carcinoma.

## Discussion

CLNM is common among esophageal SCC patients [8], [11]. Li et al. reported the pattern of thoracic SCC lymph node metastases after three-field esophagectomy, the rates of CLNM in patients with upper, middle and lower thoracic tumors were 41.6%, 33.3% and 36.4%, respectively [11]. The prognosis of patients with CLNM from stage IVa/IVb esophageal cancer is not uniform. Furthermore, patients with CLNM have significantly better survival rates than patients with solid-organ metastases [8], [12], [13]. Liu et al. compared SCC patients with enlarged cervical paraesophageal lymph nodes without any other metastatic cervical lymph nodes (CPLNM) and those with enlargement of any other cervical lymph nodes apart from the cervical paraesophageal lymph nodes (OCLNM) [8]. They concluded that CPLNM could be considered as regional spread in patients with upper thoracic SCC, which is consistent with the AJCC staging system (7^th^ edition) [6]. The above results suggest that the prognosis of esophageal cancer patients with CLNM might be better than that of patients with distant metastases. However, the prognostic significance of unilateral CLNM in terms of survival has not yet been explored in detail.

### Effects of unilateral CLNM vs. other metastases on OS

To our knowledge, this is the first large-scale study to investigate the prognosis of SCC patients with unilateral CLNM. In nasopharyngeal carcinoma patients, unilateral CLNM has been associated with better distant metastasis–free survival rates than bilateral CLNM on univariate analysis [14]. In our study, we found that the 3-year OS rate was better in patients with CLNM (29.7%) than in patients with distant organ metastases (5.7%, *p* = 0.027), which is consistent with the results reported by Nomura et al. [15]. Nomura et al. reported that the 3-year OS rates of esophageal cancer in stages III, IVa and IVb were 37.1%, 34.2% and 9.1%, respectively. Furthermore, the survival curve for stage IVa was significantly better than the curve for stage IVb [15]. In the current study, we divided our patients into three groups (group A, group B and group C). Survival rates significantly differed between the three groups (3-year OS: *p*<0.001; 3-year PFS: *p*<0.001). We next performed post hoc pairwise comparisons among the three groups. Heo et al. compared four post hoc multiple pairwise testing procedures, and concluded that the Hochberg procedure has a low false discovery rate and the highest correct decision rate among the four pairwise methods. We therefore adopted the Hochberg step-up method in the pairwise comparison, and the p value of the pairwise comparisons was set at 0.017 (0.05/3) [10]. The pairwise comparisons showed that unilateral CLNM (group B) had a similar prognosis to that of patients with regional disease (group A), and had a better prognosis than that of patients with other distant metastases (group C). This suggests that some patients with M1a disease, according to the 6th edition of the AJCC staging system (thoracic esophageal cancer patients with unilateral CLNM) can be staged as having regional disease. Both the JSED guidelines and the Chinese clinical non-operative staging of esophageal cancer consider CLNM as regional disease; however, neither have analyzed this prognostic factor in these patients in as much detail as we did [6], [7]. Thus, our results complement these clinical staging systems.

We consider that the similarity in the prognoses of thoracic esophageal carcinoma patients with unilateral CLNM and patients with regional disease is chiefly attributable to the fact that in the case of unilateral CLNM, the target for radiotherapy is one contiguous area [16], [17]. The radiation fields may not cover all tumor lesions satisfactorily in patients with other distant metastases. Furthermore, patients with unilateral CLNM may be in an earlier stage than patients with other distant metastases. Skip metastasis may be another reason influencing prognosis. Unilateral CLNM is more likely to be a skip metastasis. Prenzel et al. reported that skip metastases are associated with better 5-year survival rates and are more common in patients with cancer of the middle and upper thirds of the esophagus [18].

### Significant prognostic factors

In the current study, univariate analysis showed that pathological grade did not significantly influence the OS and PFS of patients with advanced SCC, which is in accordance with the AJCC staging system (7^th^ edition) [6]. However, inconsistent with this staging system, the prognosis of patients with primary tumors located in the upper third of the esophagus was better than that of patients with tumors located in the lower two-thirds. This may be due in part to differences in treatment strategies: all patients enrolled in the 7^th^ staging system received surgery. Among the 4628 patients enrolled in the study of the 7^th^ staging system, only 177 (4.1%) had cancer of the upper third of the esophagus [19].

In our study, radiation dose was not a significant factor on univariate analysis, which suggests that high radiation doses do not increase OS or PFS in advanced SCC patients. This result is consistent with the results of Radiation Therapy Oncology Group (RTOG) 9405 [20]. RTOG 9405 has concluded that a higher radiation dose did not result in higher survival or better locoregional control in M0 patients. The authors did not determine the reason for this lack of benefit in the high-dose arm; however, they supposed that considerable prolongation of the treatment time and the administration of a low dose of 5-FU because of toxicity may have contributed, at least in part, to this result [20].

Primary tumor length was not a significant factor on multivariate analysis. It seems that the prognostic significance of tumor length is lower in advanced-stage patients than in early-stage patients. Yendamuri et al. reported that esophageal tumor length is independently associated with long-term survival, but is not statistically significant in stage III patients [21].

Female patients had an obviously better prognosis than did male patients in our study. A previous study has revealed that female esophageal cancer patients have a survival advantage [22]. The T stage of the primary lesion also affect the prognosis. In our study, T1/2 patients had a higher survival rate than did T3 patients, and the prognosis of T3 patients was better than that of T4 patients. Increasing depth of tumor invasion is associated with the presence of lymphatic dissemination, and thus leads to an unfavorable prognosis [23]. Analysis of the PFS rates revealed the same results.

Chemotherapy regimen was a significant independent predictor of prognosis in esophageal SCC, and cisplatin plus docetaxel was the most effective regimen for concurrent chemoradiotherapy. No phase III clinical trial has thus far confirmed this result. However, van Hagen et al. reported that the rate of complete pathological response was a relatively satisfactory 49% in patients who received preoperative chemoradiotherapy for esophageal or junctional SCC [24]. The chemotherapy regimen used in their study was carboplatin plus paclitaxel (50 mg/m^2^).

### Limitations

There are several limitations of our study. First, it was a single-institution retrospective study. Second, only SCCs were included in this study because differences in morbidity and treatment strategies between different histological types may have affected the results obtained. Finally, staging was based on radiological studies. However, the specificity and accuracy of CT for detecting nodal metastases are 96.7% and 76.6%, respectively, and these values are similar to those for positron emission tomography; nodal enlargement of >10 mm is considered indicative of cancerous involvement [25]. Therefore, our results should be further evaluated in other large cohorts.

## Conclusion

In summary, we have shown that SCC patients with unilateral CLNM have a long-term survival advantage over those with other distant metastases. Unilateral CLNM can be regarded as regional disease in SCCs. The current study also revealed that clinical T stage, cervical lymph node involvement and concurrent chemotherapy modality were independently associated with the prognosis of patients with esophageal SCC.
